# Context Effects in the Judgment of Visual Relative-Frequency: Trial-by-Trial Adaptation and Non-linear Sequential Effect

**DOI:** 10.3389/fpsyg.2018.01691

**Published:** 2018-09-12

**Authors:** Xiangjuan Ren, Muzhi Wang, Hang Zhang

**Affiliations:** ^1^Academy for Advanced Interdisciplinary Studies, Peking University, Beijing, China; ^2^Peking-Tsinghua Center for Life Sciences, Peking University, Beijing, China; ^3^School of Psychological and Cognitive Sciences and Beijing Key Laboratory of Behavior and Mental Health, Peking University, Beijing, China; ^4^PKU-IDG/McGovern Institute for Brain Research, Peking University, Beijing, China

**Keywords:** probability distortion, subjective probability, frequency estimation, sequential effect, adaptation, Bayesian inference, efficient coding

## Abstract

Humans' judgment of relative-frequency, similar to their use of probability in decision-making, is often distorted as an inverted-*S*-shape curve—small relative-frequency overestimated and large relative-frequency underestimated. Here we investigated how the judgment of relative-frequency, despite its natural reference points (0 and 1) and stereotyped distortion, may adapt to the environmental statistics. The task was to report the relative-frequency of black (or white) dots in a visual array of black and white dots. We found that participants' judgment was distorted in the typical inverted-*S*-shape, but the distortion curve was influenced by both the central tendency and spread of the distribution of objective relative-frequencies: the lower the central tendency, the higher the overall judgment (contrast effect); the higher the spread, the more curved the inverted-*S*-shape (curvature effect). These context effects are in the spirit of efficient coding but opposite to what would be predicted by Bayesian inference. We further modeled the context effects on the level of individual trials, through which we found not only a trial-by-trial adaptation, but also the non-linear sequential effects that were recently reported mainly in circularly distributed visual stimuli.

## Introduction

The human perceptual system adapts to the environmental statistics from time to time (Helson, 1947; Gilchrist et al., 1999; Dean et al., 2005; Chopin and Mamassian, 2012; Gepshtein et al., 2013). For example, a lighted outdoor sign that dazzles at night may look dim in the daylight. Adaptation like this allows the human brain to use neurons of limited dynamic range to represent the immense dynamic range of physical stimuli (10^9^ for luminance, from star light illumination to intense daylight conditions). But it comes at a cost: in order to be sensitive to differences in the current environment, the mapping from physical stimuli to perception must be non-stationary. That is, a stimulus that is physically 5 times as large as a second stimulus may be perceived 10 times as large as the latter in one context and only 2 times as large in a different context. This non-stationarity can be harmless in many situations (e.g., in the perception of lightness), where only ordering information (e.g., which is brighter and which is darker) is required.

Here we investigated how the judgment of relative-frequency may adapt to the environment. For the perception of relative-frequency, adaptation can be both helpful and harmful. On one hand, relative-frequency in real life, like luminance, has a vast dynamic range that may challenge the neural system. For example, the relative-frequencies of different causes of death span six orders of magnitude (Lichtenstein et al., 1978). On the other hand, as a source of probability information, relative-frequency needs an accurate representation. Any non-stationary transformations accompanying adaptation would hurt one's ability to maximize expected gain in decision-making.

Relative-frequency differs from many sensory stimuli in its abstractness and in its finite range—from 0 to 1. What is special about relative-frequency is also its stereotyped distortion: Humans' judgment of relative-frequency, similar to their use of probability in decision-making (Tversky and Kahneman, 1992; Gonzalez and Wu, 1999), is often distorted in an inverted-*S*-shape—small relative-frequency overestimated and large relative-frequency underestimated. For example, people overestimate the relative-frequency of rare causes of death such as flood and hurricane and underestimate that of common causes such as heart disease (Lichtenstein et al., 1978; see Zhang and Maloney, 2012 for more examples). The opposite pattern, *S*-shaped distortion, was also reported (Shuford, 1961; Pitz, 1966; Brooke and MacRae, 1977; Wu et al., 2009). Zhang and Maloney (2012) found that the inverted-*S*- or *S*-shaped distortion in a variety of tasks could be well-captured by a Linear-in-Log-Odds (LLO) transformation:

$$\begin{array}{r} \lambda \left[\pi \left(p\right)\right]=\gamma \lambda \left[p\right]+\left(1-\gamma \right)\lambda \left[p_{0}\right], \end{array}$$

where *p* and π(*p*) respectively denote objective and subjective probability or relative-frequency, λ[·] denotes the log-odds transformation, $\lambda \left[p\right]=\log\frac{p}{1-p}$, and γ and *p*_0_ are free parameters that are readily interpretable. The parameter γ indicates the slope of the distortion curve, with γ < 1 for inverted-*S*-shaped distortion, γ = 1 for no distortion, and γ > 1 for *S*-shaped distortion. The parameter *p*_0_ indicates the crossover point where π(*p*) = *p*. In other words, the γ and *p*_0_ are measures respectively for the curvature and elevation of probability distortion (Gonzalez and Wu, 1999).

**Figure 1:**
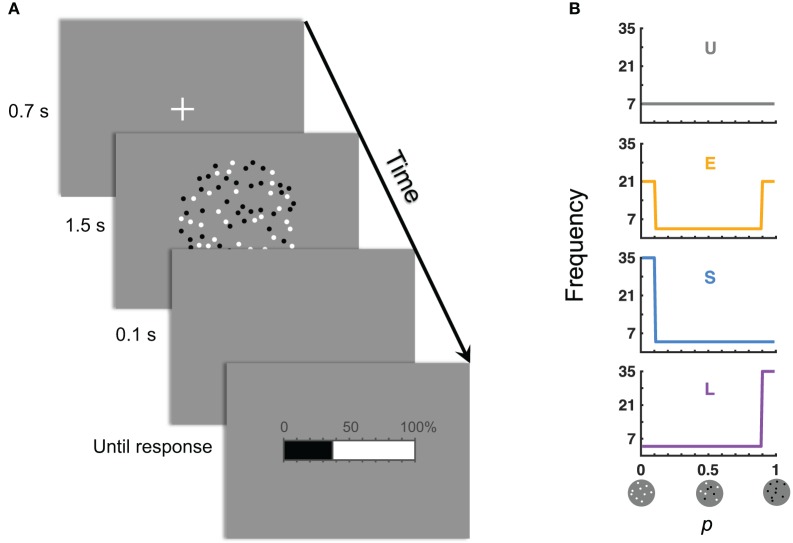
The experiment: judgment of relative-frequency. **(A)** Time course of a trial. The task was to judge the relative-frequency of the black (or white) dots in the array of black and white dots and to report the judgment on the 0–100% scale. **(B)** Distributions of the objective relative-frequencies in the four experimental conditions. The values of the objective relative-frequencies (denoted *p*) could be 0.01, 0.02, …, 0.99. Those in the ranges of [0.01, 0.10] and [0.90, 0.99] were referred, respectively, as the small and large *p*'s. “U” denotes the condition with a Uniform distribution of *p*. “E” denotes the condition where the Extreme (small and large) *p*'s dominated. “S” denotes Small *p*'s dominated. “L” denotes Large *p*'s dominated.

In our experiment, participants judged the relative-frequency of black (or white) dots among an array of black and white dots (Figure 1A). There were four conditions for the distribution of the objective relative-frequency *p* (Figure 1B). In the baseline Uniform condition, *p* was uniformly distributed between 0.01 and 0.99. The Small (Large) condition differed from the Uniform condition mainly in the central tendency of the distribution by having a disproportionally great number of very small (large) values of *p*. The Extreme condition was U-shaped (i.e., most values were extreme) and differed from the Uniform condition in the spread of the distribution.

We asked two questions. The first question is whether and how participants' judgment of relative-frequency, π(*p*), may vary with the distribution of *p*. There has been increasing evidence that adaption functions not only for sensory modalities, but also for abstract quantities such as utility (Tobler et al., 2005; Kobayashi et al., 2010; Louie et al., 2013; Khaw et al., 2017; Rustichini et al., 2017), numerosity (Burr and Ross, 2008; Cicchini et al., 2014), rate (Levitan et al., 2015), and variance (Payzan-LeNestour et al., 2016), in the form of contrast effects: the same quantity tends to be perceived larger in a context of small quantities and smaller in contrast with large quantities. Such contrast effect was also found for relative-frequency in a task similar to ours (Varey et al., 1990).

**Figure 2:**
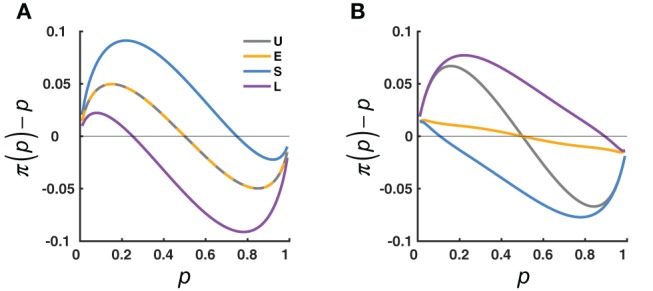
Opposing effects predicted by two influential lines of theories. The predicted deviation of the subjective from objective relative-frequency, π(*p*)−*p*, is plotted as a function of the objective relative-frequency, *p*, and compared across the four distribution conditions (U, E, S, L, color coded). **(A)** Adaptation-level theory (Helson, 1947). The π(*p*) is assumed to reflect the difference between the *p* and a reference point known as “adaptation-level”, which shifts with the average value of the distribution. Thus, π(*p*) is repelled away from the concentrated areas of *p*: compared to the U condition, there is an overall overestimation for the S condition, and an overall underestimation for the L condition; there is no difference between the U and E conditions (the interleaved dashed lines), since the two have the same adaptation-level. **(B)** Judgment as Bayesian inference (Jazayeri and Shadlen, 2010). The subjective relative-frequency, π(*p*), is assumed to be a posterior estimate that integrates the percept with the prior distribution of the objective relative-frequency. Thus, π(*p*) is attracted toward the concentrated areas of *p*: compared to the U condition, there is less overestimation of small *p*'s and less underestimation of large *p*'s for the E condition, an overall underestimation for the S condition, and an overall overestimation for the L condition.

What concerned us are not individual values but how the whole curve of π(*p*) may change with the context and what principles the changes may follow. We considered two lines of theories that provide opposite predictions for the possible context effects (see Figure 2 for the simulated predictions). One line of theories is represented by the adaption-level theory (Helson, 1947; Parducci, 1965), which assumes that the perception of a specific stimulus reflects the difference between the stimulus and an internal reference point. The value of the reference point, called “adaptation level”, is determined by the average value of the stimuli in the context. The adaptation-level theory predicts a contrast effect (Figure 2A): The Small condition, which had a lower central tendency than the Uniform condition, would lead to a higher elevation for the π(*p*) curve, while the Large condition would lead to a lower elevation than the Uniform condition. Because the adaptation level is not influenced by the spread of the distribution, the adaptation-level theory predicts no difference between the Extreme condition and the Uniform condition.

The second line of theories treats perceptual judgment as a Bayesian inference problem [see (Maloney and Zhang, 2010; Petzschner et al., 2015) for reviews]—inferring the true value of a physical stimulus (in the current experiment, relative-frequency) based on its noisy percept. To compensate for the uncertainty in the percept, the final judgment would combine the percept and prior information about the stimulus. If the prior participants used follows the distribution of *p*'s they had experienced in the experiment, their judgment would be biased toward the high-density regions of the distribution. Thus, the Bayesian inference theory predicts an assimilation effect (Figure 2B): The Small condition, which had high densities on the small end, would have a lower elevation than the Uniform condition, while the Large condition would have a larger elevation than the Uniform condition. Similarly, for the U-shaped Extreme condition, the concentration of *p*'s on the two ends would attract π(*p*)toward the two ends, that is, a steeper slope than the Uniform condition.

Our second question is how the context effects, if any, may arise from trial to trial. In our experiment, participants were never explicitly informed about the distribution of *p* and could only learn the distribution via individual trials. We modeled two processes on the level of individual trials. The first process is a trial-by-trial updating of reference point, which is a natural extension of the adaptation-level theory with the additional assumption that the adaptation level (reference point) is updated by the delta rule (Rescorla and Wagner, 1972). As the result, the reference point assigns higher weights to more recent trials and would be able to track the changes in the context.

The second process we investigated is the sequential effect, that how the stimulus or response of a precedent trial may bias the current response. A common practice to quantify the sequential effect (Pegors et al., 2015) is to regress the current response (*R*_*n*_) against the stimulus (*S*_*n*−*i*_) or response (*R*_*n*−*i*_) *i*-trial back, *i* = 1, 2, …, *m*. In this way, it is assumed that *R*_*n*_ changes linearly with *S*_*n*−*i*_ or *R*_*n*−*i*_, or, in other words, the sequential effect is linear. Most sequential effects documented in the literature are linear sequential effects (Fründ et al., 2014). Fischer and Whitney (2014), however, reported a non-linear sequential effect in the perception of orientation: the sequential effect first increases then decreases as the distance between the previous stimulus and current stimulus increases. Non-linear sequential effects were also found in the perception of motion (Alais et al., 2017), facial identity (Liberman et al., 2014) and numerosity (Cicchini et al., 2014), and in visual working memory of colors (Makovski and Jiang, 2008). We were interested in whether there were similar non-linear sequential effects in the judgment of relative frequency and how these inter-trial effects might contribute to the global context effect.

Here is a brief summary of our experimental results: we found that the judgment of relative-frequency is distorted, as typical, but the distortion curve changes with the distribution of relative-frequencies in both curvature and elevation. The observed context effect in elevation agrees with the prediction of the adaptation-level theory but opposite to that of Bayesian inference, while the effect in curvature can be accounted by neither theory but conforms to the principle of efficient coding (Attneave, 1954; Barlow, 1961; Simoncelli and Olshausen, 2001; Wei and Stocker, 2012, 2015; Burr and Cicchini, 2014; Summerfield and Tsetsos, 2015). On the level of individual trials, we found evidence for a trial-by-trial adaptation and a non-linear sequential effect, which could partly account for the observed context effects.

## Methods

### Ethics statement

The experiment had been approved by the Institutional Review Board of School of Psychological and Cognitive Sciences at Peking University. All participants gave written informed consent in accordance with the Declaration of Helsinki.

### Participants

Sixty-four students of Peking University participated (28 male, aged 18–29) and were randomly assigned into four experimental conditions, with 16 participants for each condition. All participants had normal or corrected-to-normal vision. The experiment took ~70 min and participants received 50 RMB (≈ 8 USD) for their time.

### Apparatus and stimuli

Participants were seated ~86 cm (i.e., 1.5 cm ≈ 1° of visual angle) in front of a 21.5″ iMac monitor (47.3 × 26.6 cm, 1,920 × 1,080 pixels, 60-Hz refresh rate). The display of stimuli and recording of responses were controlled by the iMac computer using Matlab and PsychToolbox-3 (Brainard, 1997; Pelli, 1997).

Stimuli on each trial were an array of black and white dots on a gray background (Figure 1A). Dots were located randomly but non-overlapped within a centered invisible circle that subtended a visual angle of 10°. Each dot subtended ~0.1°.

### Procedure and design

The task was to judge the relative-frequency of the black (or white) dots in the array of black and white dots. Half of the participants judged for the black dots and the other half for the white dots. Figure 1A shows the time course of a trial: Shortly after the onset and offset of a fixation cross, an array of black and white dots was presented for 1.5 s, followed by a horizontal bar with tick marks from 0 to 100%. Participants were asked to click on the bar to report their estimate of relative-frequency. In particular, when they moved the mouse left and right, the indicator on the bar (i.e., the boundary between the black and white regions) moved accordingly. Participants confirmed their estimates by left clicking the mouse, which terminated the trial. There was no time limit for response.

The total number of dots in a display could be 200, 300, …, 800, and the relative-frequency (denoted *p*) could be 0.01, 0.02, …, 0.99. There were four conditions as below, concerning the distribution of *p*'s across trials (Figure 1B). The Uniform condition refers to a uniform distribution of *p* on the range of [0.01, 0.99]. In the Small condition, small *p*'s had a higher density−50/99 of *p*'s were in the range of [0.01, 0.1]. In the Large condition, large *p*'s had a higher density−50/99 of *p*'s were in the range of [0.9, 0.99]. In the Extreme condition, there were 30/99 of *p*'s on each end ([0.01, 0.1] or [0.9, 0.99]). Participants' judgment is denoted π(*p*). What concerned us is how π(*p*), as a function of *p*, may differ between different distribution conditions.

Each participant only completed one distribution condition. There were 693 trials, divided into 7 blocks of 99 trials. Each participant also completed 35 practice trials prior to the formal experiment. No feedback was given during the experiment. Participants were encouraged to respond as accurate as they could.

### Simulation of theoretical predictions

#### Judgment as bayesian inference

One may apply the framework of Bayesian inference to model the judgment of relative-frequency. The original percept, *y*, is assumed to be disturbed by a Gaussian noise on the log-odds scale:

$$\begin{array}{r} \mathrm{Pr}\left(\lambda \left[y\right]|p\right)\propto \exp\left(-\frac{{\left(\lambda \left[y\right]-\lambda \left[p\right]\right)}^{2}}{2\overset{2}{\underset{noise}{\sigma }}}\right), \end{array}$$

where Pr denotes probability, *p* denotes the objective relative-frequency, λ[·] denotes the log-odds transformation, and σ_*noise*_ is a free parameter. Transforming back into the probability scale, we have:

$$\begin{array}{r} \mathrm{Pr}\left(y|p\right)\propto \frac{\exp\left(-\frac{{\left(\lambda \left[y\right]-\lambda \left[p\right]\right)}^{2}}{2\overset{2}{\underset{noise}{\sigma }}}\right)}{y\left(1-y\right)}. \end{array}$$

Suppose participants' prior for a specific distribution condition is the same as the true distribution θ(*p*), which is defined separately for the Uniform, Extreme, Small, and Large conditions as below:

() $$\begin{array}{l} \theta_{U}\left(p\right)=\frac{1}{99},\;p\in \left\{0.01,0.02,\ldots ,0.99\right\},\\ \theta_{E}\left(p\right)=\{\begin{array}{cc} \frac{30}{10}\cdot \frac{1}{99}, & p\in \left\{0.01,0.02,\ldots ,0.1\right\}\\ \frac{39}{79}\cdot \frac{1}{99}, & p\in \left\{0.11,0.12,\ldots ,0.89\right\}\\ \frac{30}{10}\cdot \frac{1}{99}, & p\in \left\{0.9,0.91,\ldots ,0.99\right\} \end{array},\\ \theta_{S}\left(p\right)=\{\begin{array}{cc} \frac{50}{10}\cdot \frac{1}{99}, & p\in \left\{0.01,0.02,\ldots ,0.1\right\}\\ \frac{49}{89}\cdot \frac{1}{99} & p\in \left\{0.11,0.12,\ldots ,0.99\right\} \end{array},\\ \theta_{L}\left(p\right)=\{\begin{array}{cc} \frac{49}{89}\cdot \frac{1}{99}, & p\in \left\{0.01,0.02,\ldots ,0.89\right\}\\ \frac{50}{10}\cdot \frac{1}{99} & p\in \left\{0.9,0.91,\ldots ,0.99\right\} \end{array}. \end{array}$$

According to Bayes' theorem, for any *q*∈{0.01, 0.02, …, 0.99}, given the percept *y* and the prior θ(*q*), the posterior probability of the stimulus being *q* is:

$$\begin{array}{r} \mathrm{Pr}\left(q|y\right)\propto \mathrm{Pr}\left(y|q\right)\theta \left(q\right). \end{array}$$

Following Jazayeri and Shadlen (2010) Bayes Least-Square model, we assumed that participants, in order to minimize the mean square error, would use the expectation of the posterior distribution as their estimate for *p*. The estimate conditional on a specific percept *y* is

$$\begin{array}{r} \pi_{y}=\underset{q}{\sum }q\mathrm{Pr}\left(q|y\right). \end{array}$$

Given that the percept *y* itself is a random variable that cannot be directly observed, we need to marginalize off *y* to obtain a mapping from *p* to the final estimate π(*p*):

$$\begin{array}{r} \pi \left(p\right)=\int \pi_{y}\mathrm{Pr}\left(y|p\right)dy. \end{array}$$

For Figure 2B, the parameter σ_*noise*_ = 1.

#### Adaptation-level theory

The adaptation-level theory does not predict the inverted-*S*-shaped distortion itself but predict how the distortion may change with the context. In our simulation for the adaptation-level theory, the π(*p*) is determined by the same equation as LLO except for the inclusion of the adaptation level *L*:

$$\begin{array}{r} \lambda \left[\pi \left(p\right)\right]=\gamma \left(\lambda \left[p\right]-\lambda \left[L\right]\right)+\left(1-\gamma \right)\lambda \left[p_{0}\right], \end{array}$$

where, as in LLO, γ and *p*_0_ are free parameters, and λ[·] denotes the log-odds transformation. The value of *L* shifts with the distribution of *p*:

$$\begin{array}{r} \lambda \left[L\right]=\eta \underset{p}{\sum }\theta \left(p\right)\lambda \left[p\right], \end{array}$$

where η is a free parameter and θ(*p*) is defined for each distribution condition as in Equation (4).

For Figure 2A, the parameters γ = 0.8, *p*_0_ = 0.5, η = 0.2 .

### Measures of distortion of relative-frequency

#### Slope and crossover point estimated from LLO

For each participant, we used LLO (Equation 1) to fit the reported relative-frequency, π(*p*) and estimated the slope parameter γ and the crossover point parameter *p*_0_.

#### Smoothed distortion curve and non-parametric measures

The γ and *p*_0_ provide a model-based summary for a distortion curve. Still, critical details of the curve may be lost due to the limitation of the model. As a complementary analysis, we smoothed the distortion curve for each participant and elicited non-parametric measures of distortion from the smoothed curve.

In particular, we smoothed π(*p*)−*p* using a kernel regression method with the commonly-used Nadaraya-Watson kernel estimator (Nadaraya, 1964; Watson, 1964; Aljuhani and Al turk, 2014):

$$\begin{array}{r} {\hat{M}}_{h}\left(x\right)=\frac{\sum_{i=1}^{m}K\left(\frac{x-x_{i}}{h}\right)y_{i}}{\sum_{i=1}^{m}K\left(\frac{x-x_{i}}{h}\right)}, \end{array}$$

where *x*_*i*_ and *y*_*i*_ (*i* = 1, 2, …, *N*) denote observed pairs of stimuli and responses, ${\hat{M}}_{h}\left(x\right)$ denotes the smoothed response at the stimulus value *x*, and *h* is a parameter that controls the degree of smoothing. The *K*(·) denotes the Gaussian kernel function

$$\begin{array}{r} K\left(z\right)=\frac{1}{\sqrt{2\pi }}\exp\left(-\frac{z^{2}}{2}\right). \end{array}$$

According to the optimal bandwidth selection algorithm by Bowman and Azzalini (1997) the optimal values of *h* for different conditions and participants ranged from 0.02 to 0.07. To avoid possible artifacts for using different values of *h*, we set *h* to be 0.03. We computed the smoothed value of π(*p*)−*p* for *p* = 0.01, 0.02, …, 0.99 based on the observations of all trials.

The curvature measure for the smoothed distortion curve was defined as the area between the curve and the zero line, which is inversely related to the γ in LLO. The elevation measure was defined as the area of the curve above the zero line minus that below the zero line, which is related to the *p*_0_ in LLO.

### Estimating sequential effects

For each participant, we performed a linear regression to estimate the possible dependence of the current response on the responses of previous trials:

$$\begin{array}{r} R_{n}=\beta_{0}S_{n}+\overset{m}{\underset{i=1}{\sum }}\beta_{-i}R_{n-i}+\beta_{C}+\varepsilon , \end{array}$$

where *R*_*n*_ denotes the current response, *S*_*n*_ denotes the current stimulus, *R*_*n*−*i*_ denotes the responses *i-trial* back, β_0_, β_−*i*_, β_*C*_ are free parameters, and $\varepsilon \left(0,\sigma_{noise}^{2}\right)$ is a Gaussian random noise term. Following LLO, we used responses and stimuli that are both in the form of log-odds, that is, $S_{n}=\lambda \left[p_{n}\right]=\log\frac{p_{n}}{1-p_{n}}$, $R_{n}=\lambda \left[\pi \left(p_{n}\right)\right]=\log\frac{\pi \left(p_{n}\right)}{1-\pi \left(p_{n}\right)}$.

It is possible that *R*_*n*_ is influenced by the previous responses *R*_*n*−*i*_ as well as by the previous stimuli *S*_*n*−*i*_. But because *R*_*n*−*i*_ and *S*_*n*−*i*_ were highly correlated (Pearson's *r* > 0.746, *p* < 0.001 for all participants) and it was not our major interest to distinguish between the influences of *R*_*n*−*i*_ and *S*_*n*−*i*_, we did not include both of them in the linear regression.

We did not assert that the sequential effects were really linear. That is, the β_−*i*_ in Equation (12) was not necessarily constant across different stimuli. In a further analysis, we estimated the value of β_−1_ as a function of *p*_*n*_ and *p*_*n*−1_ using weighted least-square regressions (WLS), denoted ${\hat{\beta }}_{-1}^{WLS}$. For the regression centered at a specific pair of (*p*_*n*_, *p*_*n*−1_), where *p*_*n*_, *p*_*n*−1_ ∈ {0.01, 0.02, …, 0.99}, the weight of trial *j* was determined by a two-dimensional Gaussian kernel function:

$$\begin{array}{r} w_{j}\left(p_{n},p_{n-1}\right)=\frac{1}{2\pi \sigma_{k}^{2}}\exp\left(-\frac{{\left(p_{j}-p_{n}\right)}^{2}+{\left(p_{j-1}-p_{n-1}\right)}^{2}}{2\sigma_{k}^{2}}\right) \end{array}$$

where σ_*k*_ denotes the span of the Gaussian kernel and was set to be 0.1, *p*_*j*_ and *p*_*j*−1_ respectively denote the objective relative-frequency of trial *j* and trial *j*−*1*, *j* = 2, 3, …, *N*. If we define

$$\begin{array}{rcl} W & = & \left(\begin{array}{cccc} w_{1}^{2}\left(p_{n},p_{n-1}\right) & 0 & \cdots & 0\\ 0 & w_{2}^{2}\left(p_{n},p_{n-1}\right) & \cdots & 0\\ \vdots & \vdots & \ddots & \vdots \\ 0 & 0 & \cdots & w_{N}^{2}\left(p_{n},p_{n-1}\right) \end{array}\right), \end{array}$$

$$\begin{array}{rcl} X & = & \left(\begin{array}{ccc} S_{2} & R_{1} & 1\\ S_{3} & R_{2} & 1\\ \vdots & \vdots & \vdots \\ S_{N} & R_{N-1} & 1 \end{array}\right), \end{array}$$

$$\begin{array}{rcl} Y & = & \left(\begin{array}{c} R_{2}\\ R_{3}\\ \vdots \\ R_{N} \end{array}\right), \end{array}$$

the coefficients of the weighted least-square regression at (*p*_*n*_, *p*_*n*−1_) could be estimated as:

$$\begin{array}{r} \left(\begin{array}{c} {\hat{\beta }}_{0}^{WLS}\\ {\hat{\beta }}_{-1}^{WLS}\\ {\hat{\beta }}_{C}^{WLS} \end{array}\right)={\left(X^{T}WX\right)}^{-1}X^{T}WY. \end{array}$$

### Modeling

We considered six models of π(*p*), which are all based on LLO but differ in two dimensions: whether to include trial-by-trial adaptation and the type of sequential effects assumed. In the equations for all models, *R*_*n*_ ($=\lambda \left[\pi \left(p_{n}\right)\right]=\log\frac{\pi \left(p_{n}\right)}{1-\pi \left(p_{n}\right)}$) denotes the current response, *S*_*n*_ ($=\lambda \left[p_{n}\right]=\log\frac{p_{n}}{1-p_{n}}$) denotes the current stimulus, *R*_*n*−*i*_ denotes the responses *i*-trial back, β_0_, β_−*i*_, β_*C*_ are free parameters, and $\varepsilon \left(0,\sigma_{noise}^{2}\right)$ is a Gaussian random noise term.

The baseline model LLO, as defined earlier in Equation (1), assumes no adaptation or sequential effects. To make its notations consistent with the other five models, we formulize it as:

$$\begin{array}{r} R_{n}=\beta_{0}S_{n}+\beta_{C}+\varepsilon . \end{array}$$

The AL model is the same as the LLO model except for the inclusion of an adaptation-level term:

$$\begin{array}{r} R_{n}=\beta_{0}\left(S_{n}-L_{n}\right)+\beta_{C}+\varepsilon , \end{array}$$

where *L*_*n*_ denotes the adaptation level on Trial *n*, which varies from trial to trial following a delta-rule learning:

$$\begin{array}{r} L_{n}=L_{n-1}+\kappa \left(S_{n-1}-L_{n-1}\right), \end{array}$$

where κ is a free parameter for learning rate. We did not formulize any model with a fixed adaptation-level term, because a fixed additional term to Equation (19) would be assimilated into β_*C*_ and thus the model would reduce to LLO (Equation 18).

The LLO-L model is the LLO model with linear sequential effects (see Equation 12). Similarly, the AL-L model is the AL model with linear sequential effects:

$$\begin{array}{r} R_{n}=\beta_{0}\left(S_{n}-L_{n}\right)+\overset{m}{\underset{i=1}{\sum }}\beta_{-i}R_{n-i}+\beta_{C}+\varepsilon . \end{array}$$

In models with linear sequential effects, the influence of *R*_*n*−*i*_ is the same β_−*i*_ for any *R*_*n*−*i*_. The LLO-NL model is the LLO model with non-linear sequential effects whose strength decreases with the distance between *R*_*n*−*i*_ and *S*_*n*_:

$$\begin{array}{r} R_{n}=\beta_{0}S_{n}+\overset{m}{\underset{i=1}{\sum }}\beta_{-i}\left(R_{n-i}-S_{n}\right)\exp\left[-\frac{{\left(R_{n-i}-S_{n}\right)}^{2}}{2\omega^{2}}\right]+\beta_{C}+\varepsilon , \end{array}$$

where ω is a free parameter. With *R*_*n*−*i*_ multiplied by $\exp\left[-\frac{{\left(R_{n-i}-S_{n}\right)}^{2}}{2\omega^{2}}\right]$, the ω determines how fast the influence of *R*_*n*−*i*_ decreases with the distance between *R*_*n*−*i*_ and *S*_*n*_.

Similarly, the AL-NL model is the AL model with non-linear sequential effects:

$$\begin{array}{rcl} R_{n} & = & \beta_{0}\left(S_{n}-L_{n}\right)+\overset{m}{\underset{i=1}{\sum }}\beta_{-i}\left(R_{n-i}-S_{n}\right)\exp\left[-\frac{{\left(R_{n-i}-S_{n}\right)}^{2}}{2\omega^{2}}\right]\\ +\beta_{C}+\varepsilon . \end{array}$$

See Table 1 for a summary of models. The parameters of the models were estimated using maximum likelihood estimates. The MATLAB function *fmincon* (with the interior-point algorithm) was used for searching for the parameters that minimized negative log likelihood. To verify that we had found the global minimum, we repeated the searching process for 300 times with different starting points. Only *R*_*n*_'s with trial number *n* ≥ 6 were fitted so that the *i* in *R*_*n*−*i*_ could take values up to 5 (*n*−*i* must be positive).

**Table 1:** Notations.

**VARIABLES OR FUNCTIONS**	
*p*	A generic value on the probability scale; objective probability or relative-frequency
λ(·)	Log-odds function of probability or relative-frequency . λ(*p*) = log(*p*/(1−*p*))
π(*p*)	Subjective probability or relative-frequency
*p*_*n*_	Objective probability or relative-frequency of Trial *n*
*S*_*n*_	Stimulus of Trial *n* in log-odds. *S*_*n*_ = λ(*p*_*n*_) = log(*p*_*n*_/(1−*p*_*n*_))
*R*_*n*_	Response of Trial *n* in log-odds. *R*_*n*_ = λ(π[*p*_*n*_]) = log(π[*p*_*n*_]/(1−π[*p*_*n*_]))
**MODEL ABBREVIATIONS**	
LLO	Linear in log-odds model
AL	Adaptation-level model
LLO-L	Linear in log-odds model with linear sequential effects
LLO-NL	Linear in log-odds model with non-linear sequential effects
AL-L	Adaptation-level model with linear sequential effects
AL-NL	Adaptation-level model with non-linear sequential effects
**MODEL PARAMETERS**	
γ	Slope of the linear transformation of log-odds
*p*_0_	Crossover point; controlling the intercept of the linear transformation of log-odds
β_0_	Coefficient for the *S*_*n*_ term
β_−*i*_	Coefficient for the *R*_*n*−*i*_ term, *i* = 1,2,…,5
β_*C*_	Coefficient for the constant term
σ_*noise*_	Standard deviation of the Gaussian noise
κ	Learning rate of the adaptation-level
**ω**	Scope-of-influence parameter of LLO-NL or AL-NL; controlling how fast the sequential effect decreases with the difference between *R*_*n*−*i*_ and *S*_*n*_

### Efficient coding analysis

In any case the *p*-to-π(*p*) mappings were different between the Uniform condition and the other three conditions, we would be interested in whether the change of mapping across conditions agrees with efficient coding. Given that the responses for the judgment of relative-frequency are limited between 0 and 1, the distribution of responses that would maximize information transfer is a uniform distribution over the range of [0, 1] (Simoncelli and Olshausen, 2001). For a specific condition (Extreme, Small, or Large), if its *p*-to-π(*p*) mapping deviates from that of the Uniform condition in the direction of efficient coding, the distribution of its observed responses should be more similar to the uniform distribution than the response distribution predicted by the mapping of the Uniform condition is.

We quantified the dissimilarity between a specific response distribution and the uniform distribution over [0, 1] using the Kullback-Leibler (KL) divergence:

$$\begin{array}{r} D_{KL}\left(response||uniform\right)=\overset{\;}{\underset{i}{\sum }}f_{r}\left(i\right)\log\frac{f_{r}\left(i\right)}{f_{u}\left(i\right)} \end{array}$$

where *f*_*r*_(*i*) and *f*_*u*_(*i*) respectively denote the probability of the response distribution and the probability of the uniform distribution in the *i*-th bin, *i* = 1, 2, …, 10, with bins evenly divided between 0 and 1. We collapsed across trials from all 16 participants of a specific condition to obtain the observed response distribution of the condition. For each condition, we simulated the response distribution predicted by the mapping of the Uniform condition (U-mapping) as follows. For the stimulus of each trial, a virtual response was generated by randomly choosing one response from the observed responses of the Uniform condition that were associated with an identical stimulus. The virtual responses for all trials formed the U-mapping predicted distribution. We repeated the simulation to generate 1,000,000 U-mapping predicted response distributions. For conditions other than the Uniform condition, efficient coding implies that *D*_*KL*_(*observed*||*uniform*)−*D*_*KL*_(*U-mapping*||*uniform*) < 0.

## Results

We chose to use non-parametric statistical tests whenever possible, because most of the variables tested were parameters estimated from models (e.g., the γ and *p*_0_ estimated from LLO) that were not necessarily normally distributed. Unless otherwise stated, the significance level of 0.05 was used. The capital letter *P* was used to denote the value of significance, in order to be distinguished from the notation of relative-frequency (*p*).

### Context effects

**Figure 3:**
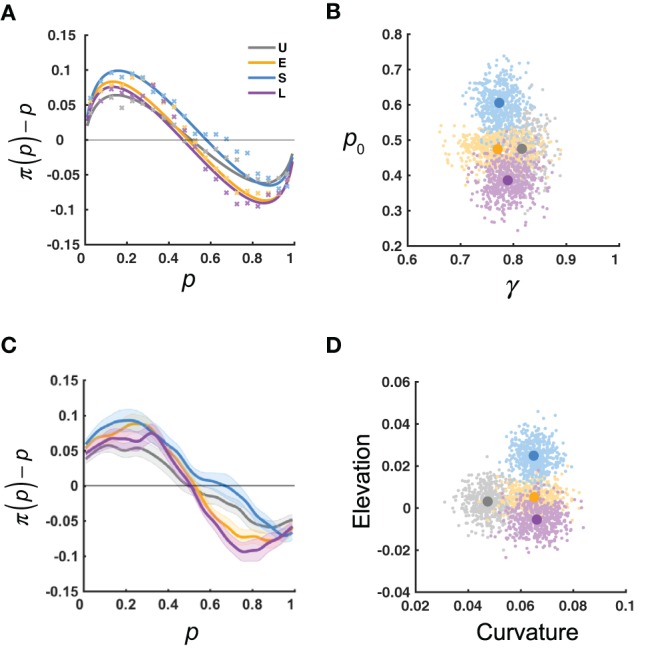
Results: measures of probability distortion. U, E, S, L denote the four distribution conditions. **(A)** π(*p*)−*p* as a function of *p*: data (xs) vs. LLO model predictions (curves), averaged across participants for each condition. Each x denotes the mean estimate for a bin of *p*, with bin size of 0.05. **(B)** Slope (γ) and crossover point (*p*_0_) measures of probability distortion estimated using the LLO model. The variability of the estimated mean γ and *p*_0_ is visualized by their bootstrap resamples, in clouds of dots. **(C)** Smoothed π(*p*)−*p* as a function of the objective relative-frequency (*p*). The π(*p*)−*p* curve was first smoothed for each participant and then averaged across participants for each distribution condition. Shadings denote 1 SE. **(D)** Curvature and elevation measures of probability distortion derived from the π(*p*)−*p* curve of **(C)** The curvature measure is defined as the area between the curve and the zero line, which is inversely related to the γ in LLO. The elevation measure is defined as the area of the curve above the zero line minus that below the zero line, which is related to the *p*_0_ in LLO. The variability of the estimated mean curvature and elevation is visualized by their bootstrap resamples, in clouds of dots. The bootstrap procedure was as follows: for each condition and each pair of parametric or non-parametric measures, we randomly sampled with replacement for 16 times from the 16 participants and computed the means of the estimated measures for the resampled participants. This procedure was repeated for 500 times to generate the 500 resamples visualized in each cloud.

We performed two lines of analyses to quantify how the distortion of relative-frequency may adapt to the environmental statistics. First, for each participant, we fitted the LLO model to the reported relative frequency, π(*p*), and estimated the slope γ and the crossover point *p*_0_ for the distortion. The predictions of the LLO model agreed well with the data (Figure 3A).

The mean γ and *p*_0_ for each distribution condition are shown in Figure 3B. Participants in all conditions had mean γ < 1, indicating the typical inverted-*S*-shaped distortion. Based on the predictions of the adaption-level theory and Bayesian inference, we were interested in whether the Uniform and Extreme conditions differed in γ and whether the four conditions differed in *p*_0_. For γ, a two-tailed Wilcoxon rank sum test showed that the difference between the Uniform condition (median 0.82) and the Extreme condition (median 0.76) failed to reach significance, *Z* = 1.26, *P* = 0.21. According to a Kruskal–Wallis test (a non-parametric equivalent of one-way ANOVA) on *p*_0_, different distribution conditions differed significantly in *p*_0_, χ^2^(3) = 12.44, *P* = 0.006, with *post-hoc* multiple comparisons (Tukey-Kramer corrected) showing the Small condition (median 0.59) had a significantly larger *p*_0_ than the Large condition (median 0.39).

In a second line of analysis, we obtained a smoothed curve of π(*p*)−*p* (Figure 3C). We can see the Small condition had the largest crossover point (i.e., the point the curve crosses the zero line) among the distribution conditions, which agrees with the findings about *p*_0_ above. Meanwhile, the Uniform condition was less curved (less deviated from the zero line) than the Extreme condition.

To characterize the differences visible in the smoothed distortion curve, we defined the curvature metric (the area between the curve and the zero line) and the elevation metric (the area of the curve above the zero line minus that below the zero line) for each participant (Figure 3D). By definition, the curvature is inversely related to γ for γ ≤ 1 and the elevation is related to *p*_0_. We performed similar tests on the curvature and elevation as we did for γ and *p*_0_. For the curvature, a two-tailed Wilcoxon rank sum test showed that the Uniform condition (median 0.050) had a significantly smaller curvature than the Extreme condition (median 0.073), *Z* = −2.28, *P* = 0.023. This difference was in the same direction as the insignificant trend in γ . According to a Kruskal–Wallis test, different distribution conditions differed significantly in the elevation, χ^2^(3) = 10.18, *P* = 0.017. *Post-hoc* multiple comparisons (Tukey-Kramer corrected) showed that the Small condition had a significantly larger elevation (median 0.025) than the Large condition (median −0.011), which was consistent with the finding above that the Small condition had a significantly larger *p*_0_ than the Large condition.

In sum, we found that both the curvature and elevation of the distortion were influenced by the statistical environment. That the Small condition had a higher elevation than the Large condition is in accordance with the prediction of the adaptation-level theory (Figure 2A) but against that of Bayesian inference (Figure 2B). In contrast, our finding that the Extreme condition had a higher curvature than the Uniform condition could not be explained by either theory. However, as we discuss later, it agrees with the principle of efficient coding.

### Sequential effects

**Figure 4:**
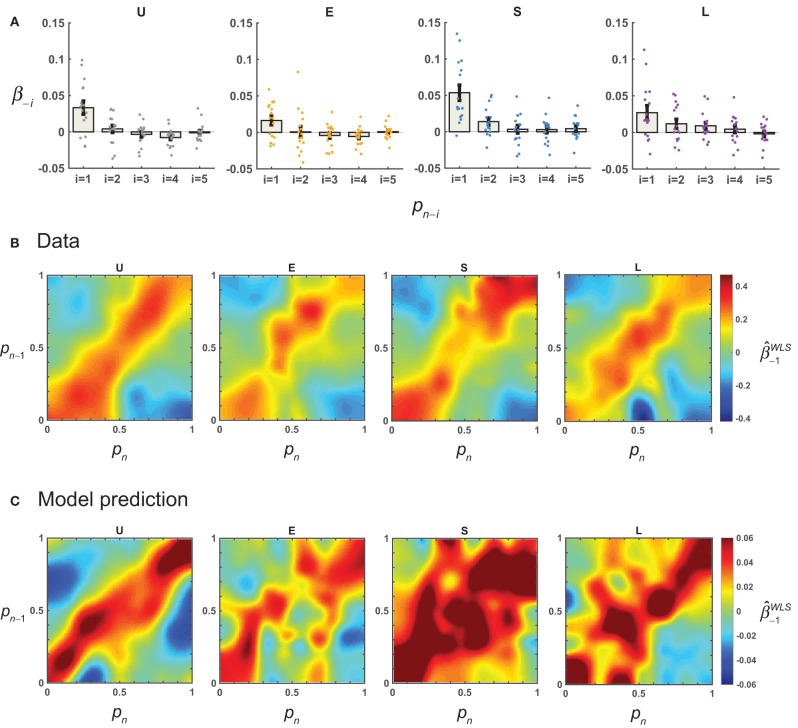
Results: sequential effects. For each participant, linear regression was performed to quantify the possible influence of the previous response on the current response (Equation 12), and how this sequential effect—as quantified by the coefficient for the previous response, β_−1_—might depend on the value of *p* on the current (*p*_*n*_) and previous trials (*p*_*n*−1_). **(A)** Sequential effects for more than one trial back. The estimated mean β_−*i*_ across participants for the responses up to five trials back is plotted for each distribution condition. Error bars denote 1 SE. Dots denote estimates for individual participants. **(B)** Sequential effects as a function of *p*_*n*_ and *p*_*n*−1_. The mean ${\hat{\beta }}_{{\;}_{-1}}^{WLS}$ across participants is plotted separately for the four conditions. Note that the closer the *p*_*n*−1_ was to the *p*_*n*_, the larger the ${\hat{\beta }}_{{\;}_{-1}}^{WLS}$. **(C)** AL-NL model's predictions for sequential effects.

We used linear regressions to estimate the possible contribution of the previous response (*R*_*n*−1_) to the current response (*R*_*n*_). For each participant and distribution condition, we first regressed *R*_*n*_ against *S*_*n*_ and *R*_*n*−1_ (Equation 12, with *m* = 1) for all trials and denoted the coefficient for *R*_*n*−1_ as β_−1_. The median β_−1_ for the Uniform, Extreme, Small, and Large conditions were respectively 0.033, 0.019, 0.033, and 0.017, all significantly greater than 0 (Two-tailed Wilcoxon rank sum tests, *P* < 0.03), indicating an attraction effect of previous response. When we extended the regressors to the responses up to five trials back, only β_−1_ was significantly different from 0 (Figure 4A). Therefore, we only considered the sequential effect up to one trial back in the subsequent analysis.

But was the sequential effect really linear? To test this, we estimated the β_−1_ as a function of *p*_*n*_ and *p*_*n*−1_ using weighted least-square regressions (see section Methods). A linear sequential effect would imply a homogeneous regression coefficient map, that is, the β_−1_would not change with the values of *p*_*n*−1_ or *p*_*n*_. Instead, the estimated β_−1_ (i.e., ${\hat{\beta }}_{{\;}_{-1}}^{WLS}$) showed a clear pattern of stimulus-dependence (Figure 4B): its value decreased as the distance between *p*_*n*_ and *p*_*n*−1_ increased. The highest values of ${\hat{\beta }}_{{\;}_{-1}}^{WLS}$ occurred on the diagonal line when *p*_*n*_ equaled *p*_*n*−1_.

This non-linear sequential effect could be well predicted by the AL-NL model (Figure 4C, see Equation 23 for the model), which assumes that the weight for the previous trial decreases with the inter-trial distance in the form of a Gaussian function.

To quantify the similarity between data and model predictions in the pattern of sequential effects, for each model we computed the correlation (Pearson's *r*) between the matrix of the mean ${\hat{\beta }}_{{\;}_{-1}}^{WLS}$ observed and that predicted by the model. There were high correlations for models assuming non-linear sequential effects (LLO-NL and AL-NL), but much lower correlations or even negative correlations for models assuming linear (LLO-L and AL-L) or none (LLO and AL) sequential effect (Table 2). In sum, the modeling analysis provided converging evidence that the sequential effect was non-linear and showed that the non-linear form we assumed in the -NL models could well capture the pattern of sequential effects in the data.

**Table 2:** Pearson's *r* between data and model predictions in the pattern of sequential effects.

**Condition**	**LLO**	**AL**	**LLO-L**	**AL-L**	**LLO-NL**	**AL-NL**
Uniform	−0.015	−0.048	−0.215	−0.217	0.773	0.668
Extreme	−0.355	−0.174	−0.126	−0.318	0.723	0.588
Small	−0.141	0.219	−0.367	0.120	0.709	0.666
Large	0.238	−0.046	0.137	0.041	0.577	0.657

### Modeling the processes underlying the context effects

What trial-by-trial processes might underlie the context effects of π(*p*), given that participants were never explicitly informed about the distribution of relative-frequencies? We modeled two processes—trial-by-trial learning of adaptation-level and non-linear sequential effect—and tested whether they contributed to a better explanation of the observed π(*p*). In particular, we constructed six alternative models (see section Methods and Table 1) to compare the assumption of dynamic adaptation-level (“AL” models) with that of constant adaptation-level (“LLO” models), and to compare the assumption of non-linear sequential effect (“-NL” models) with that of linear (“-L” models) or none (null-postfix models) sequential effect. All models were fitted to each participant's π(*p*) using maximum likelihood estimates.

#### Model comparison

To compensate for the difference in number of parameters between models, we computed the Akaike information criterion corrected for small sample-size (AICc; Akaike, 1974; Hurvich and Tsai, 1989),

$$\begin{array}{r} AICc=-2\ln\;\left(\hat{L}\right)+2k+\frac{2k\left(k-1\right)}{N-k-1}, \end{array}$$

for each participant and each model as the metric for goodness-of-fit, where $\ln\;\left(\hat{L}\right)$ denotes the log likelihood maximized, *k* denotes the number of parameters, and *N* denotes the number of trials. The lower the AICc, the better the model fit.

**Figure 5:**
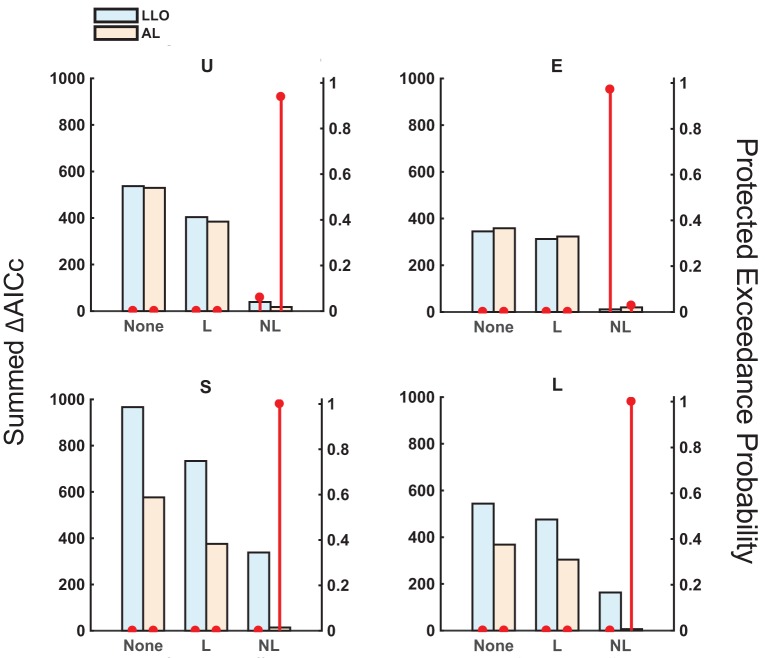
Model comparison: AICc and protected exceedance probability. The assumptions of the models differed in two dimensions: (1) without adaptation (LLO) or with adaptation (AL), and (2) without sequential effects (“None”), with linear sequential effects (“L”), or with non-linear sequential effects (“NL”). The summed AICc (bars, left axis, the lower the better) and protected exceedance probability (red dots, right axis, the probability that a specific model excels the other models considered, the higher the better) are plotted for each model and each distribution condition. For all except the E condition, the AL-NL model had an overwhelming advantage over the other models.

The best model among the six models was the AL-NL model for all distribution conditions except for the Extreme condition (where the best was LLO-NL and the second best was AL-NL), according to the AICc summed across participants (Figure 5). A group-level Bayesian model selection (Stephan et al., 2009; Rigoux et al., 2014) based on AICc suggested the same (see the red dot in Figure 5 for the protected exceedance probability, that is, the probability a specific model is better than all the other models). We can also see that the models with non-linear sequential effects outperformed models with linear or none sequential effects, other things being the same. Except for the Extreme condition, AL models fit better than LLO models. The advantage of AL over LLO models was small in the Uniform condition and even negative in the Extreme condition, probably because the distribution of relative-frequency was centered at *p* = 0.5 in these conditions, where the final adaptation-level differed little from its initial value.

#### Estimated parameters

**Figure 6:**
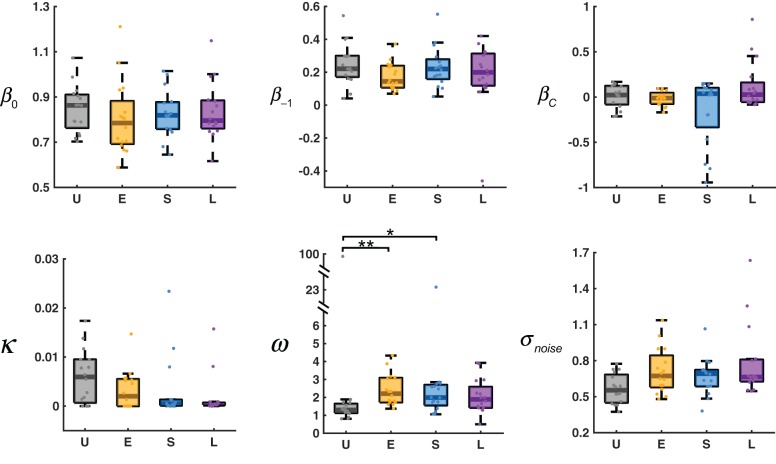
Estimated parameters for the AL-NL model. In the box plot, the middle line denotes the median estimate across participants, the bottom and top lines denote the lower and upper quartiles, and the error bars denote the 99% confidence interval. U, E, S, L denote the four distribution conditions. Dots denote estimates for individual participants. See Table 1 for the references of the parameters (β_0_, β_−1_, β_*C*_, κ , ω , σ_*noise*_). ^*^0.01 ≤ *P* < 0.05. ^**^0.001 ≤ *P* < 0.01.

The estimated parameters for the AL-NL model are shown in Figure 6. The six parameters can be divided into four categories (see section Methods and Table 1 for more details): the slope and intercept parameters that belong to the original LLO model (β_0_, β_*C*_), the learning rate of adaptation-level (κ), the parameters that control the non-linear sequential effect (β_−1_, ω), and the standard deviation of the noise term (σ).

According to Kruskal–Wallis tests separately for each parameter, different distribution conditions differed significantly only in ω , χ^2^(3) = 14.65, *P* = 0.0021. The value of ω controls how fast the sequential effect decreases with the distance between the previous response and the current stimulus. The larger the ω , the slower the sequential effect decreases with distance. In the limiting case of ω → ∞ , the sequential effect would be stimulus-independent, equivalent to a linear sequential effect.

The median value of ω for the Uniform condition (1.296) was the smallest among the four conditions, significantly smaller than those of the Extreme condition (2.209) and Small condition (1.989), and marginally significantly smaller than that of the Large condition (1.890, *P* = 0.062), according to *post-hoc* multiple comparisons (Tukey-Kramer corrected) following the Kruskal–Wallis test. Why should ω —the parameter that controls how fast the sequential effect decreases with the difference between adjacent trials—differ between conditions? We noticed that the average distance between two adjacent trials in the Uniform condition (1.936) was significantly smaller than those of the other three conditions (2.945, 2.316, 2.317, respectively for Extreme, Small, Large), according to a Kruskal–Wallis test (χ^2^(3) = 53.22, *P* < 0.001) and *post-hoc* multiple comparisons (*P* < 0.001). It seems the choice of ω adapted to the average between-trial distance of the environment or statistics alike.

## Discussion

Relative-frequency, similar to probability, is an abstract quantity that does not rely on the physical energy of stimuli and requires the involvement of higher cognition. It differs from many kinds of abstract quantity such as numerosity and utility in that it is naturally bounded between 0 and 1. Here we investigated how the perception of visual relative-frequency may change with the environmental statistics. As typical, the judgment π(*p*) was distorted as an inverted-*S*-shaped curve of the objective relative-frequency *p*. We found two context effects concerning the π(*p*) curve. The first one was about the elevation of the curve: The lower the central tendency of the distribution of *p*, the greater π(*p*)−*p*. This is consistent with the contrast effect widely reported in the adaptation literature including that specially for relative-frequency (Varey et al., 1990), as well as with the prediction of the adaptation-level theory (Helson, 1947).

We also found a second context effect concerning the spread of the stimuli: the more dispersed the distribution of *p*, the more curved the inverted-*S*-shape of π(*p*). Had π(*p*) not changed across contexts, when there were more *p*'s on the two ends as in the Extreme condition, there would be more π(*p*) 's on the two ends as well. An increase in curvature in the Extreme condition implies a change of *p*-to-π(*p*) mapping so that π(*p*) could be more evenly distributed between 0 and 1. Such effect cannot be explained by the adaption-level theory, for which adaptation equals to the adjustment of a single reference point. What may be relevant is Parducci (1965) range-frequency theory for categorical responses, where observers are supposed to adjust their responses to balance the number of responses in each category. However, the range-frequency theory is not directly applicable, because the responses in our task were not categorical but continuous.

**Figure 7:**
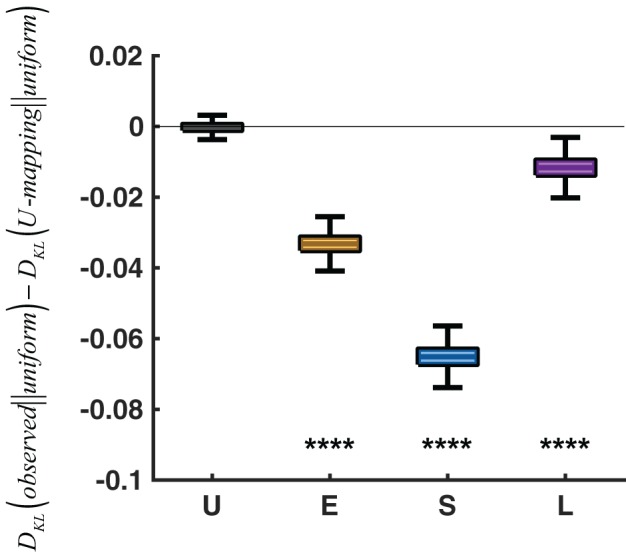
Evidence for efficient coding. *D*_*KL*_(*observed*||*uniform*) denotes the KL divergence from the uniform distribution over [0, 1] to the observed response distribution of a specific condition. *D*_*KL*_(*U-mapping*||*uniform*) denotes the KL divergence from the uniform distribution to the response distribution predicted by the *p*-to-π(*p*) mapping of the Uniform condition. *D*_*KL*_(*observed*||*uniform*)−*D*_*KL*_(*U-mapping*||*uniform*) is plotted for each condition, with the Uniform condition serving as a sanity check (i.e., the difference should be 0). A negative difference implies that the *p*-to-π(*p*) mapping adopted by a specific condition is closer to efficient coding than applying the mapping of the Uniform condition to the condition. In the box plot, the middle line denotes the median across 1,000,000 simulations, the bottom and top lines denote the lower and upper quartiles, and the error bars denote the 99% confidence interval. ^****^P < 0.0001.

The two context effects together suggest adaptations of relative-frequency that go beyond the adjustment of a single reference point. It echoes neurophysiological studies where neurons adjust to both the central tendency and spread of the stimulus distribution in utility (Kobayashi et al., 2010) as well as in sensory responses (Dean et al., 2005). Such adaptations of *p*-to-π(*p*) mappings are in the spirit of efficient coding (Attneave, 1954; Barlow, 1961; Simoncelli and Olshausen, 2001), to the goal of maximizing the discrepancy between different stimuli. In particular, we tested whether the response distribution observed in a specific condition (Extreme, Small, or Large), compared with that predicted by the mapping of the Uniform condition, was closer to the optimal response distribution. Given the limited range of responses in the task, the response distribution that maximizes information transmission is a uniform distribution over [0, 1] (Simoncelli and Olshausen, 2001). Figure 7 shows that the response distributions of the Extreme, Small, and Large conditions were significantly closer to the uniform distribution than those predicted by the mapping of the Uniform condition were (*P* < 0.0001). That is, if participants had used the same *p*-to-π(*p*) in these conditions as in the Uniform condition, the distribution of their responses would have been less optimal than the observed, in the perspective of efficient coding.

According to Petzschner et al. (2015), Bayesian inference provides a parsimonious explanation for the biases in magnitude estimation for length (Laming, 1997), time (Jazayeri and Shadlen, 2010), distance, and angle (Petzschner and Glasauer, 2011). One of the phenomena explained was the regression effect (also known as regression-to-mean) that small magnitudes are overestimated and large magnitudes underestimated. This account may even apply to the inverted-*S*-shaped distortion of probability and relative-frequency. Unfortunately, Bayesian inference fails to predict either of the two context effects we found when we manipulated the distribution of relative-frequency systematically. That being said, it is still possible that Bayesian inference may play a role, though non-dominant, in the judgment of relative-frequency. As we discuss later, the non-linear sequential effects probably reflect a compensation for uncertainty that resembles Bayesian inference.

We went further to model how the context effects may arise trial by trial and identified two trial-by-trial processes. First, compared with a constant adaptation-level, a trial-by-trial adjusted adaptation-level could better explain the observed contrast effect in elevation. When the adaptation-level is updated after each trial as a weighted average of the previous adaptation-level and the current stimulus, the value of the adaptation-level would gradually approach the central tendency of the stimulus distribution.

One mystery is the lack of adaptation in the Extreme condition. We conjecture that it is partly due to the larger distance between two adjacent trials in the condition (2.945) than those of the other conditions (1.936, 2.316, 2.317). There is evidence that adaptation may stop to work when the discrepancy between trials are too large (Levitan et al., 2015). A second possibility for the lack of adaptation concerns the bimodal distribution of objective relative-frequencies used in the Extreme condition. While adaptation for a unimodal distribution leads to an adaptation level close to the mode of the distribution, similar adaptation for a bimodal distribution may result in an adaptation level falling between the two modes and thus representative of neither mode. For this reason, the perceptual system may adopt a different strategy for bimodal or multimodal distributions. These possibilities need to be tested in the future.

Second, we found a non-linear sequential effect: the current response was biased toward the response on the previous trial, with the size of the bias well captured by a Derivative-of-Gaussian (DoG) function of the inter-trial distance. Sequential effects had been widely reported in perceptual and cognitive tasks (Fründ et al., 2014), which can be rationalized in the framework of Bayesian inference as ways of compensating for sensorimotor uncertainty (Körding and Wolpert, 2004; Jazayeri and Shadlen, 2010; Petzschner and Glasauer, 2011; Cicchini et al., 2012; Raviv et al., 2012). If the percept for the current trial were independent of those of the precedent trials in random noises, combining the current percept with previous responses appropriately would allow one to achieve a less varied response than using the current percept alone. In practice, the weight for a precedent trial was often modeled as a constant so that the response would be a linear combination of the current percept and previous responses. However, it is recently found in the perception of orientation (Fischer and Whitney, 2014) that the weight received by a precedent trial is not a constant; instead, it decreases with the distance between the current and precedent trial in the feature space. In discovering so, Fischer and Whitney (2014) plotted the judgment error of the current trial as a function of the orientation difference between the previous and current stimuli and obtained a telling DoG-shaped curve that implies decreasing weight for the precedent trial as the inter-trial distance increases. Similar DoG-shaped curves and thus non-linear sequential effects have been identified in the perception of motion (Alais et al., 2017) and facial identity (Liberman et al., 2014) and in visual working memory of colors (Makovski and Jiang, 2008).

Except for numerosity (Cicchini et al., 2014), all the non-sequential effects found so far were for circularly distributed stimuli via plotting judgment error as a function of inter-trial distance. The same visualization can hardly qualify as a test for the linearity of sequential effects in non-circularly distributed stimuli (though see Figure 4 for clues of non-linearity), because different stimuli would be associated with different distributions of inter-trial distances. In the case of relative-frequency, the difficulty of visualization was increased by the systematic biases inherent in the judgment. We used modeling methods to overcome the difficulty: We constructed models assuming different forms of sequential effects, among which models with non-linear sequential effect fit best to the observed π(*p*).

The non-linear sequential effects were conjectured to be a mechanism that helps to keep visual stability across space and time (Fischer and Whitney, 2014). Our finding of similar non-linear sequential effects in the abstract quantity relative-frequency, along with that of numerosity (Cicchini et al., 2014), suggests a more general mechanism than the previously theorized. In fact, it implies a bifurcation: when the previous stimulus is close to the current stimulus, the current response merges the two; when the previous stimulus is far from the current stimulus, the current response simply dismisses the previous one. Such bifurcations have been widely observed in the group decision of animals (Couzin, 2009), in the competition between neurons (Nichols and Newsome, 2002), and in the integration of information from multiple sensory modalities (Wozny et al., 2010).

We found that the non-linear sequential effect could adapt to the distribution of *p*. In the winning AL-NL (or LLO-NL) model, the strength of the bias toward the previous trial is controlled by two parameters: a scaling factor β_−1_ and the scope-of-influence ω . The value of ω but not β_−1_ had significant differences between different distribution conditions.

It would be interesting to see what factors may influence the non-linear sequential effects. Fischer and Whitney (2014) found that the size of the non-linear sequential effects in the perception of orientation would decrease with the spatial or temporal proximity between trials. Whether the non-linear sequential effects found in the judgment of relative-frequency follow similar principles is unknown. Whether non-linear sequential effects may give way to linear sequential effects under certain circumstances is also an empirical question.

To conclude, human judgment of relative-frequency adapts to the environmental statistics trial by trial toward the direction of maximizing the discrepancy between different stimuli. Between trials there are also non-linear sequential effects that may help to reduce the variability of response.

## Author contributions

XR and HZ designed the experiment. XR performed the experiment. XR, MW, and HZ analyzed the data and wrote the manuscript.

### Conflict of interest statement

The authors declare that the research was conducted in the absence of any commercial or financial relationships that could be construed as a potential conflict of interest.
